# Bcl-2 protein in diffuse large B-cell lymphoma

**DOI:** 10.1590/S1516-31802011000100011

**Published:** 2011-01-06

**Authors:** Viroj Wiwanitkit

**Affiliations:** I MD, PhD. Professor, Wiwanitkit House, Bangkhae, Bangkok, Thailand.; II MD, PhD. Adjunct professor, Department of Internal Medicine, Universidade Federal de Juiz de Fora (UFJF), Juiz de Fora, Minas Gerais, Brazil.; III MD, PhD. Attending physician, Department of Pathological Anatomy, Faculdade de Medicina da Universidade de São Paulo (FMUSP), São Paulo, Brazil.; IV MD, PhD. Associate professor, Department of Hematology, Faculdade de Medicina da Universidade de São Paulo (FMUSP), São Paulo, Brazil.; V MD, PhD. Associate professor, Department of Statistics, Universidade Federal de Juiz de Fora (UFJF), Juiz de Fora, Minas Gerais, Brazil.; VI MD. Attending physician, Department of Hematology, Faculdade de Medicina da Universidade de São Paulo (FMUSP), São Paulo, Brazil.; VII MD, PhD. Titular professor, Department of Hematology, Faculdade de Medicina da Universidade de São Paulo (FMUSP), São Paulo, Brazil.

Dear Editor,

I read the recently published paper on Bcl-2 with great interest.^1^ Hallack Neto et al. concluded that “Bcl-2 protein was expressed in 37% of the high-risk DLBCL patients, without any difference between the ABC-like DLBCL and GCB-like DLBCL cases.^1^” Indeed, the clinical value of determining Bcl-2 in B-cell lymphoma is still controversial. However, there are some problems in this work. The retrospective cohort design is not a common approach. There is also no risk classification from the analysis. The small number of subjects in this work might not be statistically acceptable for any conclusion.
