# Supplementary material for: Bcl-2 protein in diffuse large B-cell lymphoma
Source: Sao Paulo Med J. 2011 Jan 6;129(1):54. doi: 10.1590/S1516-31802011000100011 (PMC10865907; doi:10.1590/S1516-31802011000100011)
Supplement: Related texts

# Reply: RESPONSE TO LETTER TO THE EDITOR

**Authors:** Abrahão Elias Hallack Neto, Sheila Aparecida Coelho Siqueira, Frederico Luiz Dulley, Alfredo Chauobah, Marcelo Belesso, Rosaura Saboia, Milton Artur Ruiz, Dalton Alencar Fischer Chamone, Juliana Pereira

To the Editor

We would like to thank you very much for your attention to and comments on our trial published in issue 128(1) of this journal. As we ourselves acknowledged in the last paragraph of the discussion, the retrospective design of this trial and the small number of patients should be seen as questionable features and, therefore, our results need to be confirmed in a prospective study with more patients. However, despite the low statistical power of retrospective studies, the most important point is that such studies should be performed in order to raise questions and hypotheses for which proof may subsequently be obtained in trials with greater statistical power, in the way provided by prospective controlled trials.

Concerning the observation about the absence of risk classification from the analysis, we would like to clarify that, as described in the Methods section, all the patients enrolled in our trial were classified as intermediate-high and high risk according to the International Prognostic Index (IPI).
